# Developing a patient decision aid for the treatment of women with early stage breast cancer: the struggle between simplicity and complexity

**DOI:** 10.1186/s12911-017-0505-6

**Published:** 2017-08-01

**Authors:** W. Savelberg, T. van der Weijden, L. Boersma, M. Smidt, C. Willekens, A. Moser

**Affiliations:** 1 Oncology Centre, Maastricht University Medical Center, P. Debyelaan 25, 6229 HX Maastricht, The Netherlands; 2 School for Public Health and Primary Care (CAPHRI) Maastricht University, Universiteitssingel 40, 6229 ER Maastricht, The Netherlands; 3 Department of Family Medicine, Maastricht University, Universiteitssingel 40, 6229 ER Maastricht, The Netherlands; 4 Department of Radiotherapy, Maastricht University Medical Center, (MAASTRO clinic) Dr. Tanslaan 12, 6229 ET Maastricht, The Netherlands; 5 SBOH (Foundation for vocational training in family medicine), P. Debyelaan 25, 6229 HX Maastricht, The Netherlands; 6 Zuyd University of Applied Sciences, Nieuw Eyckholt 300, 6419 DJ Heerlen, The Netherlands

**Keywords:** Shared decision making, Patient decision aid, Early stage breast cancer, Alpha testing

## Abstract

**Background:**

A patient decision aid (PtDA) can support shared decision making (SDM) in preference-sensitive care, with more than one clinically applicable treatment option. The development of a PtDA is a complex process, involving several steps, such as designing, developing and testing the draft with all the stakeholders, known as alpha testing. This is followed by testing in ‘real life’ situations, known as beta testing, and then finalising the definite version.

Our aim was developing and alpha testing a PtDA for primary treatment of early stage breast cancer, ensuring that the tool is considered relevant, valid and feasible by patients and professionals.

**Methods:**

Our qualitative descriptive study applied various methods including face-to-face think-aloud interviews, a focus group and semi-structured telephone interviews. The study population consisted of breast cancer patients facing the choice between breast-conserving therapy with or without preceding neo-adjuvant chemotherapy and mastectomy, and professionals involved in breast cancer care in dedicated multidisciplinary breast cancer teams.

**Results:**

A PtDA was developed in four iterative test rounds, taking nearly 2 years, involving 26 patients and 26 professionals. While the research group initially opted for simplicity for the sake of implementation, the clinicians objected that the complexity of the decision could not be ignored. Other topics of concern were the conflicting views of professionals and patients regarding side effects, the amount of information and how to present it.

**Conclusion:**

The development was an extensive process, because the professionals rejected the simplifications proposed by the research group. This resulted in the development of a completely new draft PtDA, which took double the expected time and resources. The final version of the PtDA appeared to be well-appreciated by professionals and patients, although its acceptability will only be proven in actual practice (beta testing).

**Trial registration:**

NTR TC 5721.

**Electronic supplementary material:**

The online version of this article (doi:10.1186/s12911-017-0505-6) contains supplementary material, which is available to authorized users.

## Background

Women with early stage breast cancer can often choose between two options with comparable outcomes: either breast-conserving therapy (BCT), including radiation therapy, or mastectomy, with or without radiation therapy [1]. Some patients are offered neo-adjuvant systemic treatment to enable or facilitate BCT. In addition, patients increasingly have the option of reconstructive surgery of the breast after a breast removal, or even a breast-conserving operation. The outcomes of these treatments are comparable with regard to life expectancy, yet not with regard to cosmetic results, long-term side effects or treatment burden. As there is more than one clinically valid treatment option, shared decision making (SDM) is becoming increasingly important. SDM has been defined as ‘an approach where clinicians and patients share the best available evidence when faced with the task of making decisions, and where patients are supported to consider options, to achieve informed preferences’ [2].

Our aim is to implement SDM behaviour and for that purpose we designed a multifaceted implementation strategy [3]. In this paper we will focus on one of the ingredients of the implementation strategy, the Patient Decision Aid (PtDA). A PtDA can support the SDM process [4, 5]. It provides information about the disease, the possible treatment options, the pros and cons of each option, and sometimes value elicitation statements. It is increasingly recognised that using a PtDA in the communication between surgeons and women facing early stage breast cancer increases women’s knowledge about the options and their satisfaction with the decision, while reducing decisional conflict [5, 6].

**Fig. 1:**
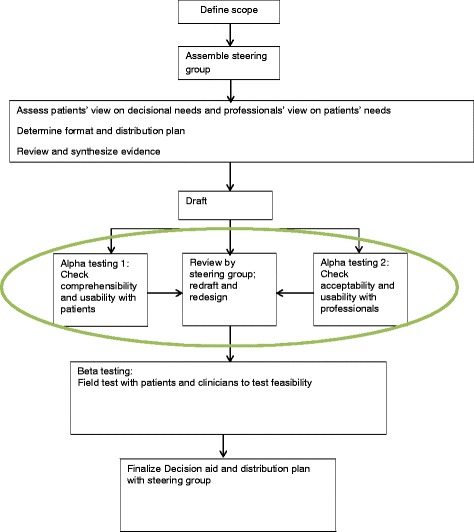
Model of development process for patient decision aids

Developing a PtDA is a time-consuming and complex process which requires the involvement of many different professionals as well as patients [4]. It needs to be done carefully, with a fully documented development process to ensure the validity and reliability of the PtDA. PtDAs can have significant impact on choices patients make. Poor quality PtDAs may cause harm to patients [4, 7, 8]. In addition, clinicians who disagree with the content of the PtDA are unlikely to use it or encourage their patients to do so [4] and will thus hamper clinical implementation. On behalf of the International Patient Decision Aids Standards (IPDAS) group, Coulter et al. [4] described quality criteria for the development of PtDAs, one of which is alpha testing, that is co-creating a draft in an iterative process among experienced patients and professionals (Figure 1) [9]. It involves patients who have faced the decision in question in the past, and clinicians working for the target population [10].

We are aware of 11 PtDAs described in the literature to support women with early stage breast cancer in deciding between breast removal and breast-conserving treatment [11–21]. Alpha testing has not been reported for any of these PtDAs. In the beta testing phase, the usability of three PtDAs was tested among patients, and acceptability of two PtDAs was tested among professionals [6, 11, 13, 15, 18]. Among the 11 existing PtDAs there was only one Dutch version. This PtDA was outdated and not used by Dutch professionals. The other PtDAs could not be easily adapted because of the differences in guidelines and care processes between countries. We wanted to ensure that we developed a PtDA that professionals want to use and patients appreciate, and that helps the latter to fully understand the options when making a decision. We chose to conduct an extensive alpha test as this seemed the optimal phase for both patients and professionals to influence the functionality and content of the PtDA, and to pave the way for clinical implementation. We believe that patient participation in the alpha testing phase is closest to co-creation. The potential impact that patients can have seems stronger during the alpha testing than the beta testing phase, as there will only be room for minor adjustments in the latter phase.

Alpha testing addresses usability, comprehensibility and acceptability aspects. Usability is defined as ‘the extent to which a product is used by a specific group to achieve certain goals effectively, efficiently and satisfactorily in a particular context [22]. Recommendations for usability testing include running many small tests with no more than five users per test round [23]. Comprehensibility has been defined as ‘the way the information is understandable’ and was especially tested among patients [24]. Acceptability, tested only among the professionals, has been defined as ‘adequate to satisfy a need, requirement or standard’ and refers to ratings regarding the comprehensibility of components of a PtDA, its length, the amount of information, balanced presentation of information about options, and overall suitability for decision making [25].

In the Netherlands, the first PtDA for this topic was developed in 2001 [16]. Although it showed positive results in an experimental setting regarding the quality of the decision-making process, patient satisfaction, doctor-patient relationship and health outcome, it has not been implemented in the daily practice of surgeons. In 2009, another PtDA, developed along with the clinical practice guideline on surgical treatment of early stage breast cancer, was published on the Dutch government’s patient portal (https://www.kiesbeter.nl/). Although it was freely available to both patients and professionals from this portal, this PtDA was not implemented in daily practice either. Most professionals and patients were not even aware of its existence. For sake of implementation we opted for simplicity and developed a draft PtDA with brief, easy to read and concise information, inspired by the one-page option grids used by clinicians during clinical encounters [26] The information was based on the existing but out-dated PtDA provided on the government’s patient portal [16], as well as the Dutch clinical guidelines (Oncoline) on breast cancer, additional literature and expert opinion. Our first draft was developed in a simple PowerPoint-like presentation by the research team, following the checklist of the IPDAS [8]. During the development process, this first draft evolved into a website including interactive elements to provide tailored information to each patient (Table 1). The PtDA consists of brief but comprehensive information about early stage breast cancer, the treatment options, the pros and cons of each option, and value elicitation statements.

**Table 1:** Overview of the draft PtDA's per round

The first draft PtDA was built in the format of a PowerPoint-like presentation. It included two surgical treatment options, survival rates, side effects, all pros and cons of the treatments, pictures of surgical results, and value elicitation statements. The second draft was built as a website. We used the same content and same number of pictures and graphs to present risks. At the end of the PtDA it showed a summary of the patient’s response to the value clarification statements. The final structure of the PtDA was established in the third draft. It is a website including interactive elements to provide tailored information to individual patients. Patients gain access into the website with a personal login code. The homepage enables them to personalise the PtDA by using a prescription pad they receive from their clinician. The content consists of the treatment options, including neo-adjuvant therapy and breast reconstruction, pros and cons, side effects and value elicitation statements.	

We hypothesised that alpha testing with patients and professionals would help ensure the validity and reliability of the development process and thus would stimulate future ownership and implementation of the PtDA. In this paper, as an illustrative example, we outline in detail the process of developing the drafts of the PtDA together with patients, patient advocates and professionals. The aim of this study was to develop, alpha test and improve a PtDA for surgical treatment of early stage breast cancer and ensure that usability, comprehensibility and acceptability of the tool itself met the standards and requirements of professionals and patients.

## Methods

### Design

We developed a draft PtDA and conducted a qualitative descriptive study to alpha test and iteratively improve the PtDA. The iterative and participatory approach involved patients, patient advocates and health professionals from all clinical disciplines (surgeons, radiotherapists, oncologists) within breast cancer care.

### Setting

The first draft was developed by a research team, consisting of a general surgeon, an implementation scientist, a clinical epidemiologist, a researcher on patient-centred care and a medical student, together with members of the dedicated breast cancer team of Maastricht University Medical Center (MUMC+, Maastricht, the Netherlands), which treats about 250 newly diagnosed breast cancer patients a year. Professionals from various Dutch hospitals and the national network of breast cancer teams were invited to take part in the alpha testing.

### Participants in the alpha testing procedure

#### Patients

The patients were recruited via staff of the MUMC+ breast cancer team and the Dutch Breast Cancer Association. Inclusion criteria for the alpha testing phase allowed the participation of patients diagnosed with early stage breast cancer who understood Dutch and who had already undergone surgical treatment. We assumed that these patients would be less likely to experience stress and anxiety, due to being exposed to a draft PtDA, than patients who had yet to start treatment. They were included using purposive sampling to reach sufficient diversity in terms of educational level and breast cancer experience (Table 2). All participants provided written consent for their participation.

**Table 2:** Characteristics of patients participating in the interviews, reported for each of the four test rounds

1 interviews on decision aid: PowerPoint presentation									
Patient no.	1	2	3	4	5	6	7	8	9
Educational level^a^	4	4	4	4	2	2	1	1	2
Type of treatment^b^	c	e,d,c,g	c	c	b,h	c	c	c	c,f,g
2 Focus group interview on decision aid: website									
Patient no.	10	11	12	13	14	15	16		
Educational level^a^	2	2	3	3	3	4	3		
Type of treatment^b^	c,f,g	b,e	d,f	d,f	c,f,g	a,d,e,f.h	c		
3 Think-aloud interviews on decision aid: personalised website									
Patient no.	17	18	19		20				
Educational level^a^	4	1	3		2				
Type of treatment^b^	a,d	c	c		c				
4 Telephone interviews on decision aid: personalised website									
Patient no.	21	22	23		24	25	26		
Educational level^a^	2	2	4		4	3	3		
Type of treatment^b^	a,c,g	a,c,g	a,d,e,i,g		d,h,e,f,g	c,f,g	c,f,g		

^a^1: lower level education; 2: intermediate level education; 3: higher education; 4: university degree

^b^a: neo-adjuvant chemotherapy; b: breast-conserving surgery followed by full mastectomy; c: breast-conserving therapy; d: mastectomy; e: radiotherapy; f: chemotherapy; g: hormonal therapy; h: reconstruction; i: immunotherapy

Recruitment for the first usability and comprehensibility round yielded nine breast cancer patients diagnosed and treated approximately 1 year prior to participation, recruited via MUMC+ professionals. The patients were found via the consultations, when visiting the outpatient clinic in their follow-up phase. Recruitment for the second round yielded six new patients, recruited by the Dutch Breast Cancer Association from its members. We placed a call on their website, indicating the inclusion criteria. In the third round, four new patients diagnosed with breast cancer within 1 year prior to participation were again recruited through professionals from the MUMC+ Oncology Centre. Finally, recruitment for the fourth round yielded six more patients via the Dutch Breast Cancer Association, through a message on their website.

#### Professionals

Twenty-six health care professionals were invited and they all participated in the alpha test. They were recruited by members of the development team, using purposive sampling to achieve diversity in terms of disciplines and hospitals (Table 3). The professionals participating in the alpha testing included oncologic surgeons, radiation oncologists, medical oncologists, and nurses from eight different hospitals with dedicated breast cancer teams, from a radiotherapy clinic, and professionals from the Dutch Breast Cancer Association. Professionals involved in the process of developing the first draft PtDA were excluded from the other rounds in the alpha test.

**Table 3:** Characteristics of professionals participating in the interviews, reported for each of the four test rounds

1 Written comments and cognitive interviews on decision aid: PowerPoint										
No. of interview	1	2	3	4	5	6	7	8		9
Type of professional^1^	a	a	a	c	c	c	d	e		f
Hospital^2^	MUMC+	AMC	LUMC	LUMC	MUMC+	MUMC+	MC	MUMC+		MUMC+
2 Consultation on decision aid: website										
No. of interview	10	11	12							
Type of professional^1^	d	a	c							
Hospital^2^	MC	MUMC+	MUMC+							
3 Think-aloud interviews on decision aid: personalised website										
No. of interview	13	14	15		16	17				
Type of professional^1^	e	a	b		c	d				
Hospital^2^	MUMC+	MUMC+	MUMC+		MUMC+	MC				
4 Telephone interviews on decision aid: personalised website										
No. of interview	18	19		20	21	22	23	24	25	26
Type of professional^a^	a	a		a	a	a	a	a	a	g
Hospital^b^	JBZ	CZE		TWZ	CWZ	LUMC	AMC	AMC	AM	-

^1^a: oncologic surgeon; b: medical oncologist; c: specialised breast care nurse; d: radiation oncologist; e: plastic surgeon; f: health educator; g: staff of Dutch Breast Cancer Association

^2^ *MUMC+* Maastricht University Medical Center, *AMC* Amsterdam Medical Center, *LUMC* Leiden University Medical Center, *JBZ* Jeroen Bosch Hospital, *MC*:MAASTRO clinic, *CZE* Catherina Hospital Eindhoven, *TWZ* Elisabeth-Tweesteden Hospital Tilburg, *CWZ* Canisius Wilhelmina Hospitals, *AM* Alexander Monro Breast Cancer Clinic

### Ethics

The study was approved by the MUMC+ ethics committee (No.14–05-42). Handling of personal data was in accordance with the Dutch Personal Data Protection Act and Medical Research (Human Subjects) Act. All participants provided written informed consent, and all data were processed confidentially and anonymously.

### Data collection

**Fig. 2:**
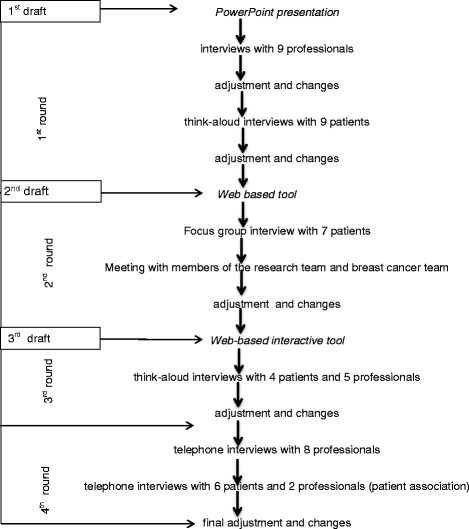
Flowchart representing the cyclic and iterative development process including the alpha testing

Data were collected between September 2012 and June 2015 using different techniques, such as think-aloud interviews, a focus group, and semi structured telephone interviews (Fig. 2).

#### Alpha testing among patients

For the first round, the researcher made individual appointments with participating patients in their home environment or at the hospital. Patients were then shown the PowerPoint prototype. A think-aloud method was used: patients were asked to go through the PtDA, and encouraged to immediately express any comments on the PtDA. After the exercise was completed, the researcher asked a few additional questions such as: ‘What is your general impression?’ and ‘What did you particularly appreciate?’ The think-aloud interviews were audiotaped and lasted between 30 and 60 min.

After a web-based PtDA had been developed, participants received a web address and a login code in the second round. They were asked to go through the website carefully, reading the content and navigating all pages. Two researchers led a two-hour focus group interview, held at the office of the Dutch Breast Cancer Association, using a structured interview technique. During the session, participants were asked to give their views on the content, navigation and layout of the PtDA in an interactive manner (Additional file 1). This session was audiotaped.

In the third round, the participants were invited to the MUMC+ Oncology Centre to view the latest web-based, interactive draft. The patients were asked to give comments while using the improved and personalised PtDA, again using the think-aloud method. The interviews, which lasted between 45 and 60 min, were audiotaped.

Finally, in the fourth round, we sent the patients a web address and a login code, and asked them to go through the website at home. After 1 week we conducted a semi-structured telephone interview. Questions related to general appreciation, what information was found relevant or missing, and navigational qualities of the website. These interviews lasted between 20 to 30 min, and were audiotaped.

#### Professionals

In the first round, each of the professionals read the PtDA individually, without the researcher being present. Researcher and professional then met and discussed problematic topics while scrolling through the PtDA, with the researcher taking notes.

For the second round, we organised a review session with members of the research and breast cancer team from MUMC+. The aim was to determine whether the current draft met the basic requirements for further testing. One of the researchers took notes.

In the third round, the professionals reviewed the PtDA using a think-aloud method. The interviews were audiotaped.

In the fourth round, professionals of dedicated breast cancer teams, as well as professionals working for the Dutch Breast Cancer Association, received a login name and a password. They were asked to review the PtDA. After the review, they were interviewed by telephone, while field notes were taken.

## Analyses

Data were analysed using a directed content analysis with three main categories – usability, comprehensibility and acceptability – as an analytical framework. A coding tree was developed for these three main categories. We then divided the main categories into smaller subcategories derived from the literature (Table 4) [23–25]. Later in the analysis process, we also worked inductively, as new subcategories emerged. Analysis took place in an iterative process: interviews and analyses were alternated if modifications and improvements to the successive drafts of the PtDA between each round made it necessary to collect additional information.

**Table 4:** Components of alpha testing

		Definition	Criteria
Patients	Comprehensibility [24]	The way the information is understandable	• The information on this website is described clearly. • The language used on this website is easy to grasp (simplicity). • The information on this website is easy to understand.
Patients & Professionals	Usability [23]	The extent to which a product is used by a specific group to achieve certain goals effectively, efficiently and satisfactorily in a particular context	• The system keeps users informed about what is going on. • The system speaks the users’ language, with words, phrases and concepts familiar to the user. • A clearly marked ‘emergency exit’ to leave the unwanted state without having to go through an extended dialogue. • Users should not have to wonder whether different words, situations, or actions mean the same thing. • A design which prevents a problem from occurring. • The users’ memory load is minimised by showing objects, actions and options. • Instructions for use of the system should be visible or easily retrievable whenever appropriate. • Content should not include information which is irrelevant or rarely needed. • Provide help and documentation if needed.
Professionals	Acceptability [25]	Suitable to meet a need, a requirement or standard	• The information is understandable. • The information is experienced as complete. • The length of the information is adequate. • There is a balanced presentation of information. • Overall, the website is suitable.

## Results

### Table 5 gives an overview for each round of the comments and how they were addressed

#### Round 1 (September 2012 – December 2013)

##### 4.1.1.1.First draft of the PtDA

The first draft PtDA, built in the format of a PowerPoint-like presentation, was meant to raise awareness of the two surgical treatment options. It included survival rates, the occurrence of the most important side effects, other pros and cons of the treatments, pictures of surgical results and value elicitation statements. Survival rates and side effects were given in percentages and presented as graphs. Value clarification consisted of five statements, all with five answering categories (totally disagree, somewhat disagree, neutral, somewhat agree, totally agree). The position of the value elicitation statements was deliberately placed at the start of the PtDA, as this was thought to be an important motivating eye-opener for the patients. Patients could choose to navigate by constantly clicking the forward button or clicking on one of the tabs. Clicking all the buttons in every slide resulted in lots of duplicate information.

**Table 5:** Comments on the prototypes per round

Round 1 (PowerPoint)	Patients	Professionals	Changes
General	Positive attitude, helping patients to ask the right questions. Navigation problems	Critical, unclear about the added value and time required to use it.	A web based PtDA was developed.
Numerical data	Unrealistic numerical data.	Nurses thought the numerical data were unrealistic.	As numerical data were evidence based, no changes were made at this point.
Value elicitation	Preferred the value elicitation at the end of the PtDA.	Preferred the value elicitation at the beginning and at the end of the PtDA.	Value elicitation at the beginning was removed.
Narrative terms in risk communication	Lacked the use of narrative terms.	Preferred the use of narrative terms.	Narrative terms were avoided.
Round 2 (first web based)	Patients	Professionals	Changes
General	Easy to read, simple to use.	No additional value.	A new web based PtDA was build.
Data on side effects and long term complications	Probability data as well as level of complaints were too optimistic.		Probability data were presented in a different way.
Extensiveness of information	Missed information on: neo-adjuvant therapy, hormonal therapy, breast reconstruction.	Missed information on heredity, neo-adjuvant therapy and breast reconstruction.	All proposed information was added.
Value elicitation	Offered no added value in this form.	Statement were too simplistic.	Statements and structure was changed.
Round 3 (second web based)	Patients	Professionals	Changes
General	Information, figures and photographs were comprehensible.	Information was relevant and the PtDA was seen as useful.	
Data on side effects and complications	Questioned description as well as level of side effects on radiotherapy.	Some questions related to their own discipline.	Data and rates were rechecked. Eventually no changes were made.
Round 4 (Final web based)	Patients	Professionals	Changes
General	Easy accessible, useful, clear.	Concise, compact and complete.	
Data on recovery after breast reconstruction	Information was too optimistic.		As this information was evidence based no changes were made.

### Comments

#### Patients

Patients’ general opinion about the first draft was positive. It was seen as useful in empowering them to ask the right questions when visiting the outpatient clinic. Nevertheless, they mentioned several aspects that could be improved:

1. They felt that the estimates on side effects were unrealistic. In their perception, more patients experience side effects than was indicated by the research-based numbers in the PtDA.
2. The position of the value elicitation statements at the start of the PtDA was questioned. They experienced these questions as confusing and did not see the purpose of these questions in the PtDA. Patients wanted to read about the facts and figures first, before being confronted with questions on their values.
3. Patients thought the risk communication was not very clear, especially where narrative terms such as ‘rarely’ and ‘few’ were used to indicate side effects of radiotherapy.
4. All patients regarded the navigation of the PtDA as difficult. They did not always know where to click and consequently slides were overlooked, resulting in important information being missed.

*‘What does this term mean, “rarely”? I mean is that 10% of the patients, less or more? Reading this, I don’t feel well informed. I have to guess, that does not feel right.’ (Patient 1.3)*

#### Professionals

The professionals’ general opinion of the PtDA was that they were cautious about its feasibility and usefulness. They doubted the ability of patients to be involved in such a difficult decision, in view of additional aspects like chemotherapy and breast reconstruction, which were only touched upon briefly. With regard to acceptability, the professionals were sceptical about implementation. They were unclear about the added value and the time required to use the PtDA. The professionals disagreed about the optimal amount of information, and which information was essential for decision making. They worried about several aspects:

1. Nurses in particular thought that the evidence-based data, for example on complication rates, were invalid. They referred to their experience of rates varying between hospitals.
2. There was also resistance to using numeric probability data. The professionals advocated the use of terms like ‘rarely’ and ‘few’ because it would prevent patients from feeling distressed.
3. According to the professionals most patients already have a preference before they are informed about the options. The professionals therefore recommended presenting the value elicitations statements twice, at the opening and end of the PtDA. This would make the PtDA more interactive and could help the patient correct possible misperceptions.

*‘Within the short time I have available for each patient, I don’t think I can manage to go through such a decision tool as well.’ (Professional 1.2)*

### Changes to the first draft

In response to the navigation problems in the PowerPoint draft, we developed a website. The website consisted of four different sections: general information, information on breast-conserving therapy, information on mastectomy, and a summary of pros and cons of each treatment. With regard to the position of the value elicitation statements, no consensus could be reached among the professionals. In view of the patients’ disapproval, the value elicitation statements at the opening of the PtDA was removed while the statements at the end remained. Estimates about side effects after radiotherapy were added and presented in an easy-to-read figure.

### Round 2 (December 2013 – August 2014)

#### Second draft of the PtDA

The second draft was built as a website. We used the same content and same number of pictures and graphs to present risks. At the end of the PtDA a summary of the completed value elicitation statements appeared. Navigation was much more simple, this prevented duplication of information.

### Comments

#### Patients

The new patients participating in this round indicated that the PtDA was attractive, easily readable, with clear language, and simple to use. The following comments were made:

1. They thought that the information on radiation therapy was too concise, with short-term and long-term effects insufficiently presented. Probability data were viewed as too optimistic, as in their perception patients experienced more side effects than the PtDA suggested.
2. Patients wanted more information on neo-adjuvant therapy and hormonal therapy. In addition, they found the information on breast reconstruction incomplete, as breast reconstruction treatments were listed but without any further explanation.
3. They also thought that the statements at the end of the PtDA, which were meant as a value elicitation tool, offered no added value and did not support the decision making process.

*‘The figures on residual damage of radiation therapy are too low. That’s because there’s been little research into this. Patients’ experience shows that the real figures are much higher.’ (Patient 2.2)*

#### Professionals

The professionals had the following comments:

1. They thought the PtDA lacked information on heredity, information on neo-adjuvant therapy, and the possibilities for a reconstruction.
2. The statements in the value elicitation did not meet the professionals’ standards because the statements did not reflect the complexity of the considerations that the women had to deal with. The professionals thought that the attributes towards the decisions were presented too simplistically.
3. They thought that the PtDA did not provide all the information that should be available to enable a sound decision. None of them saw any additional value of the PtDA with regard to the quality of decision making.

*‘If you’re going to implement this decision aid, I’m not going to use it. If I’m to use a decision aid for patients, it will have to add something to the quality of the care I provide, and this decision aid does not do that.’ (Professional 2.1)*

### Changes to the second draft

Some of the professionals involved in the development had recently attended a presentation about a PtDA for prostate cancer treatment, developed by a company of industrial designers specialising in the development of PtDAs (Zorgkeuzelab). Since the professionals were enthusiastic about the company’s approach and did not intend to adopt the current draft, it was decided to build a new website in collaboration with the company. The resulting PtDA included interactive elements to provide tailored information to each patient. The structure was simplified and easier to navigate. Contents were made more reader-friendly, with easy-to-read charts, and the content was expanded to include information on heredity, neo-adjuvant therapy and breast reconstruction. Finally, we validated the value elicitation statements. We identified attributes of the treatment options which affects the patient’s preference through interviewing patients and we asked professionals about frequently asked questions during consultation. Based on both sources, we developed crucial questions to elicit values in choosing between options.

### Round 3 (august 2014 – February 2015)

#### Third draft of the PtDA

The final structure of the PtDA was established in the third draft. It is an interactive and personalised website co-designed by a company specialising in PtDA development. Members of the MUMC+ breast cancer care team provided additional content. To prevent overload of information a ‘prescription pad’ was designed, enabling the professionals to prescribe a personalised PtDA. Each prescription contains the website address and a unique login code. On each prescription, the professional ticks the treatment options that are indicated for the individual patient according to the tumour board. Once the patient has logged in, the home page shows a brief explanation of the purpose of the PtDA along with a short video clip explaining SDM and the use of the PtDA by a surgeon and a patient. The homepage has two links ‘Your diagnosis’, and ‘Summary’. The first link enables the patient to personalise the patient decision aid, by clicking the different buttons for treatment options like they are ticked on the prescription pad. By doing so the patient ensures that she will only encounter information on options relevant to her. In addition to this general part, there are three tabs: Surgical Treatments, Breast Reconstruction and Chemotherapy. The structure of each tab is similar: first ‘Information’, second ‘Value elicitation statements’ and finally ‘Your preference’. The last two are intended to be completed, printed and taken along to a consultation with a clinician. The value elicitation includes open text boxes for patients enabling them to write down their thoughts about their preference.

### Comments

#### Patients

Patients indicated that the information as well as the figures and photographs were comprehensible. The following comments were made:

1. Patients questioned the description of side effects following radiation; they felt that the fatigue was more severe than described. They appreciated the graphs because they made the estimates more transparent.

*‘I love the way the graphs clarify the information. They look great and are very illustrative.’* (Patient 3.1)

#### Professionals

In general, the professionals thought the information in the PtDA was relevant. They believed that patients would use the PtDA at home to review the information they had already received verbally at the consultation.

The professionals also confirmed that the value elicitation was useful and helpful in making a choice. Still one type of comment remained:

1. The professionals had some questions about facts and figures within their own discipline. For instance: there were some ambiguities concerning re-operation rates after breast-conserving surgery.

*‘I think it can be very useful for the patient, enabling them to take enough time to look at everything, prior to their visit to the doctor. And be able to look at things again later.’ (Professional 3.1)*

### Changes to the third draft

Numeric information about re-operations after breast-conserving surgery was added. Medical illustrations of the breast, axilla and upper body were added.

### Round 4 (march 2015 – June 2015)

#### Fourth draft of the PtDA

Only minor details were altered.

#### Patients

The patients thought the PtDA would be very useful and found it easily accessible. Information was viewed as being understandable and described clearly, and the patients appreciated the medical illustrations.

Only one comment was left:

1. The information on breast reconstruction options, regarding the duration of the surgery and the recovery time, emotional stress, social impact and time investment, was perceived as too brief and optimistic.

*‘What is lacking is some information about the duration, the size, the recovery time after the operation. It’s not just a matter of side effects, but it’s also the effort to be invested, the burden.’ (Patient 4.2)*

#### Professionals

The professionals mentioned that the content of the PtDA was concise, brief yet complete, and that the structure was appealing. They only had some minor textual comments on the percentage of radiotherapy after mastectomy, as recent research had shown that the percentage of women treated with post-mastectomy radiotherapy was higher than indicated in the PtDA.

*‘What is lacking is that ablation is frequently followed by radiotherapy; after breast amputation patients are often given radiotherapy if there’s a positive central node.’ (Professional 5.4)*

### Changes to the fourth draft

Although requested, no further information on breast reconstruction options, regarding the duration of the surgery and the recovery time, emotional stress, social impact and time investment, was incorporated in the PtDA. Percentages of radiation therapy were discussed among the development team, but in view of the different opinions it was decided not to adjust the percentages for the time being.

## Discussion

This article outlined the process of the development and alpha testing of a PtDA in an iterative process on the complex primary treatment decision for women with early stage breast cancer. The iterated process in ongoing data collection and alternating the PtDA has proved to be a well-chosen way to involve end users and to improve the PtDA [27]. In this study professionals and patients exerted significant influence on the PtDA’s content, structure and lay-out. Patients had highly valuable views, sometimes conflicting between patients but also with professionals, about the presentation of relevant risks and values, for which we succeeded to find a common denominator that was still comprehensible for lower-educated patients. We spent much time in trying to achieve consensus between the patients and professionals on the content and format of the PtDA. The research team and the professionals involved in the development discussed the impact of all of the comments after each test round. If there were uncertainties, literature was reviewed and clinicians in relevant disciplines were asked for input. After thorough considerations the research team and professionals decided whether or not to adjust the content. We could not honour all of the sometimes conflicting recommendations of the participants, mostly due to lack of scientific evidence. This resulted in a kind of balance between the input of different stakeholders, with professionals being more dominant in defining the final content on risk communication and pros and cons of treatments, while patients were more dominant in defining the value elicitation statements.

Being engaged in the alpha testing increased the professionals’ sense of ownership, especially after the professional PtDA designers had contributed to the process of development. The consequence of working with the designers was shared ownership of the PtDA, helping to ensure content maintenance and regular updates, but also implying a loss of public accessibility of the PtDA due to a code required to login.

It was challenging to reach consensus among the professionals with regard to data on risks and outcomes. Data collection on risks such as complication rates and side effects receive less attention in trials, so we lack robust details. Professionals quite often disagreed with the estimates, arguing for instance that complication rates at their hospital differed from the average data reported in the PtDA. This is an interesting issue that resonates with the growing culture of public reporting of specific hospital performance indicators. We wanted to develop a generic PtDA, which can be used across the Netherlands, so the probabilities were based on data from the NABON Breast Cancer Audit (NBCA) and the Netherlands Comprehensive Cancer Organisation (IKNL). Where Dutch data were not available, we used the international literature. Professionals involved in the alpha testing argued that the data, such as re-operation or post-mastectomy radiation rates, differed from their own hospital performance indicators, which sometimes showed better results compared to the generic data used in the PtDA. Members of the research team explained that using generic data meant the PtDA could be used across the Netherlands. If they were not convinced, the professionals involved in the development contacted them to discuss this issue in greater depth. As colleagues with the same initial doubts, this helped create understanding. In the end, the professionals involved in the alpha test were satisfied with the standardisation of risk estimates, using exact estimates including ranges. Agreeing with the content of a PtDA is generally viewed as a facilitator for the adoption of the PtDA [4]. All the relevant professionals were involved in three of the four test rounds, assuring that every relevant discipline could give an opinion on the content of the PtDA. To ensure that breast cancer surgeons agreed on the final estimates used, only surgeons were involved in the final test round.

With regards to the content in terms of risks and benefits, a few points of difference were identified between the professionals and the patients. A continuously recurring disagreement concerned the side effects of radiotherapy. Although not supported by scientific evidence, patients felt that health problems after radiation, especially in the longer term, were more frequent than indicated in the PtDA. They based their opinion on their own experience and stories from other patients. However, the lack of scientific evidence prevented us from adjusting the facts and figures in the PtDA. Although we could have used personal stories to endorse the severity of side effects experienced by patients, after thorough consideration the research team and the involved professionals chose not to do so given the lack of consensus on the value of personal stories [28].

Another difference of opinion between patients and professionals was the position of the value elicitation statements within the PtDA. According to the professionals, most patients already have a treatment preference before they are informed about the options. The professionals therefore thought it would be helpful to complete the value elicitation twice, prior to reading the information on risks and benefits and after completing the PtDA. This could help patients correct possible misperceptions. The patients, on the other hand, thought this was confusing. It is obvious that patients require sufficient knowledge of options to realise that certain values are relevant [29]. There is not yet clear evidence whether it is more helpful to identify considerations first and then present the options that match these considerations, or to present all options prior to the value elicitation. Patients’ opinion and the lack of evidence convinced us to present the value elicitation at the end of the PtDA.

During the development process it became clear that the clinicians who had to adopt the PtDA opposed the short PowerPoint format that the research group initially developed. The research group had opted for simplicity (brief and compact), inspired by the existing ‘option grid’ on breast cancer [26] that focuses on surgical options. Option grids are one-page leaflets with a summary table to enable rapid comparison between options, structured by the patients’ frequently asked questions. They are designed for use during clinical encounters, with the additional advantages of easier design and dissemination and lower costs. The clinicians objected to such a short version as it ignored the complexity of the decision. They insisted on developing an extensive and expensive PtDA, which not only included the surgical options, but also the adjuvant and neo-adjuvant chemotherapy and breast reconstruction options. The implication of this was that we had to extend our first draft with about 20 slides, which was too much to handle in PowerPoint, so we switched to a web-based format. The clinicians argued that although a web-based PtDA takes time to develop and is expensive and difficult to use during the clinical encounter, it offers more opportunities to provide the comprehensive information required to make complex decisions. Not all treatment options mentioned above are indicated for all patients with early-stage breast cancer. To still incorporate a degree of simplicity, our web-based PtDA offers the option of personalising information to the indicated treatment options only, to enable patients to minimise the information overload.

To ensure the quality of the qualitative research, we assessed its trustworthiness. Trustworthiness criteria are credibility and transferability [30]. In our study, credibility was ensured by taking sufficient time, that is 2 years, to develop the PtDA and gather data about professionals’ and patients’ requirements regarding usability and acceptability, as well as in-depth data on comprehensibility of the content of the PtDA. Participants were invited and encouraged in several rounds to express their thoughts and concerns. A coding list consisting of criteria on acceptability, usability and comprehensibility was used to categorise the findings, which were then discussed in the research team. We gathered data by means of written comment, think-aloud interviews, focus group discussions, group discussions and telephone interviews; this was then independently analysed by two members of the team, after which interpretations were compared. Different researchers were involved in data collection, and multiple experts on breast cancer, PtDA developers and researchers were involved in the development process. Different sources were used to ensure a wide variety of patient participants, working closely with the Dutch Breast Cancer Association, and these were interviewed at different points in their treatment trajectory.

It could be a limitation that the majority of the participants were above 50 years of age. The occurrence of breast cancer in young patients is relatively low, so access to this age group is limited. In addition, with regard to young women diagnosed with breast cancer heredity causes are more frequent, resulting in strong indications for a mastectomy instead of breast conserving treatment. This means that, there is no preference sensitive choice at stake and no indication to use this PtDA.

## Conclusion

We developed a PtDA with end users in four iterative rounds. We focused on alpha testing involving patients and professionals. Although the final product was much more extensive and complex than had been initially planned, we succeeded in building a solid PtDA that was appreciated by patients and professionals alike, including the Dutch Breast Cancer Association. The development process we chose was thorough, with the involvement of many participants who could contribute to the adaption of the PtDA. The alpha testing has taken time and therefore made the development of a PtDA a prolonged and expensive process. Such a process, however, turned out to be necessary to examine all the thoughts and needs of patients, and for the professionals to examine acceptability and gain a sense of ownership, thereby facilitating clinical implementation.

We expect that presenting personalised treatment options in the PtDA will improve the uptake by patients which could subsequently enhance patients’ knowledge and increase their confidence about the decision. Although many stakeholders were included in the extensive alpha testing, and clinicians in the last test round were impressed by the content of the PtDA, there is no guarantee that such a PtDA with generic data will be adopted by every breast cancer team in the Netherlands. But the fact that in the end all the involved patients, professionals and the Dutch Breast Cancer Association accepted and appreciated the PtDA could facilitate implementation.

We are currently investigating outcomes and implementation issues in a pilot implementation study [3].

## Additional file

Additional file 1: Questions focus group. (DOC 42 kb)

## Abbreviations

BCT: Breast-conserving therapy
IKNL: Netherlands Comprehensive Cancer Organisation (Integraal Kankercentrum Nederland)
IPDAS: International Patient Decision Aids Standards
MUMC +: Maastricht University Medical Center+.
NABON: National Breast Cancer Platform (Nationaal Borstkanker Overleg Nederland)
NBCA: NABON Breast Cancer Audit
PtDA: Patient Decision Aid
SDM: Shared Decision Making
