# Supplementary material for: Developing a patient decision aid for the treatment of women with early stage breast cancer: the struggle between simplicity and complexity
Source: BMC Med Inform Decis Mak. 2017 Aug 1;17:112. doi: 10.1186/s12911-017-0505-6 (PMC5540178; doi:10.1186/s12911-017-0505-6)
Supplement: Additional file 1: — Questions focus group. (DOC 42 kb) [file 12911_2017_505_MOESM1_ESM.doc]

**Appendix 1**

Interview guide focus group

What was your first impression with regard to the PtDA?

Content of the PtDA

- What did you think about het content of the PtDA?
- Which information did you find the most relevant?
- Which information did you find the least relevant?
- What did you think about the content of the value elicitation?
- What are the improvements we have to make concerning the content?
- Which information did you miss?
- Are there any subjects you would like to add to the PtDA?

Navigation

- How did you experience the navigation of the website?
- How fast could you find all required information?
- Which information was hard to find?
- Which information was easy to find?

Amount of information

- What was your impression of the amount of information per page?

General

- Do you have any suggestion on how to improve the navigation?
- Do you have any suggestions on how to improve the content?
- Do you have any other suggestions to improve the website overall?
